# Hospital Wing Opening Sparks Antimicrobial Resistance in Wastewater Microbial Community Within the First Twelve Months

**DOI:** 10.3390/microorganisms14020285

**Published:** 2026-01-26

**Authors:** Laura Lohbrunner, Claudia Baessler, Elena Becker, Christina Döhla, Nina Droll, Ralf M. Hagen, Niklas Klein, Nico T. Mutters, Alexander Reyhe, Ruth Weppler, Manuel Döhla

**Affiliations:** 1 Institute for Hygiene and Public Health, Medical Faculty, University of Bonn, 53127 Bonn, Germany; 2 Department of Microbiology and Hospital Hygiene, Bundeswehr Central Hospital Koblenz, 56072 Koblenz, Germany; 3 Department of Hospital Pharmacy, Bundeswehr Central Hospital Koblenz, 56072 Koblenz, Germany; 4 Hospital Management, Bundeswehr Central Hospital Koblenz, 56072 Koblenz, Germany; 5 Institute of Medical Microbiology, Immunology and Parasitology, Medical Faculty, University of Bonn, 53127 Bonn, Germany

**Keywords:** antimicrobial resistance, hospital wastewater, intensive care unit, wastewater microbiome, ESKAPE pathogens, *Pseudomonas* spp., antibiotic resistance emergence, clinical antibiotic use, environmental resistome, wastewater surveillance

## Abstract

Antimicrobial resistance (AMR) in hospital wastewater is a recognized public health concern, yet the dynamics of its emergence remain poorly understood. This study aimed to characterize the quantitative and qualitative changes in the microbial community of a newly built internal medicine intensive care hospital wing following the start of patient treatment. Wastewater samples were collected regularly from eight relevant sites, including seven patient-associated locations within the intensive care ward and the central sanitary sewer where all effluent converged. Culture-based analyses targeted the “ESCAPE-SO” bacterial and fungal groups (“Enterococci”, “Staphylococci”, “Candida”, “Acinetobacter”, “Pseudomonas”, “Enterobacteriaceae”, “Stenotrophomonas”, “Others”). Comparisons were made between a 12-month pre-operation period (only flushing every 72 h to prevent contamination of the drinking water system) and the first 12 months of patient treatment. The results showed a significant increase in mean bacterial concentrations from 53 [0–349] CFU/mL before patient treatment to 8423 [3054–79,490] CFU/mL during patient treatment (*p* = 0.0224) with a particular focus on *Pseudomonas* spp. as the dominant genus. Resistance against all four main antibiotic classes of the WHO AWaRe essential “watch” list (carbapenems, third-generation cephalosporins, broad-spectrum penicillin and ciprofloxacin) emerged within the first twelve months and depended on the amount of prescribed antibiotics and the number of patients treated. These findings indicate that hospital activity drives rapid development of antimicrobial resistance in wastewater microbial communities, highlighting the critical role of clinical antibiotic use in shaping environmental resistomes. This study provides quantitative evidence that resistance can emerge within months of hospital operation, emphasizing the need for early monitoring and targeted interventions to mitigate the spread of AMR from hospital effluents into broader environmental systems.

## 1. Introduction

Antimicrobial resistance (AMR) represents a major challenge to public health worldwide. In 2016, the United Nations General Assembly launched a political declaration in which it acknowledged that “is the greatest and most urgent global risk” [1]. Infectious diseases, including AMR, were still listed in the “Top 10 Challenges for Public Health in 2024” [2]. The United Nations General Assembly launched an updated political declaration in 2024, which sets out specific targets [3]: for human health, deaths from AMR bacteria are to be reduced by 10% until 2030 compared to the 4.95 million cases in 2019; at least 60% of all countries should finance AMR action plans; in human medicine, at least 70% of all antibiotics prescribed should come from the World Health Organization’s (WHO) “Access” group; 100% of all countries should equip medical facilities with basic water supply, sewage and waste disposal, and hygiene, and 90% of all countries should provide medical facilities with an infection prevention and control program. In parallel, 40 research areas are to be promoted by 2030 in order to mitigate the expansion of AMR [4].

Hospital wastewater serves as reservoir for a diverse array of antibiotic-resistant bacteria (ARB) and antimicrobial resistance genes (ARGs). Various studies have shown that hospital wastewater is highly contaminated with ARB and ARGs [5,6,7] and that the composition of the wastewater microbiome differs significantly in settings with varying antibiotic consumption [8,9,10].

Community wastewater is affected by hospitals, whose wastewater regularly contains antibiotic residues excreted by patients or disposed of via the sewage system [11].

Although hospitals use significantly less antibiotics overall than the outpatient sector [12], they predominately use broad-spectrum antibiotics with a higher risk of resistance (in particular antibiotics on the WHO AWaRe essential “watch” list [13]). These antibiotics are often administered in an inpatient setting, as they are used as a broad-spectrum treatment for severe infections and can usually only be administered intravenously [14]. Antibiotic stewardship programs can help ensure targeted consumption and reduce the risk of resistance [15,16,17]. However, as long as antibiotics remain available as effective weapons, their use will continue [18]. To further reduce the development of resistance, new and alternative therapeutic approaches such as phage therapy are needed [19].

AMR in hospital wastewater is a recognized public health concern, yet the dynamics of its emergence remain poorly understood. While previous studies have documented the prevalence of ARB in hospital wastewater [20,21,22], there is a paucity of research focusing on the temporal dynamics of resistance development within these microbial communities. However, opportunities to investigate these processes under controlled, pre-operational conditions are rare: since hospital beds are expensive to operate, they are put into service as soon as possible after construction is completed. That means that it is not possible to achieve a sufficiently long observation period before a new ward goes into operation. In addition, new wards are usually connected to an existing wastewater system, meaning that wastewater from several wards is discharged together. It is therefore difficult to generate unbiased data on the development of a wastewater microbiome because it becomes mixed with other discharge points.

Understanding how hospital operations influence the emergence and proliferation of AMR is crucial for developing effective strategies to mitigate its spread. Due to special circumstances, this study was able to monitor a newly built intensive care unit over a period of 12 months before it was occupied by patients but with drinking and wastewater systems already in operation. Subsequently, the development of the wastewater microbiome could be observed over a further 12 months during patient operation.

This study therefore aims to characterize the quantitative and qualitative changes in the microbial community of a newly built internal medicine intensive care unit (ICU) wing following the start of patient treatment.

## 2. Materials and Methods

### 2.1. Technical Data on the Intensive Care Ward

**Figure 1:**
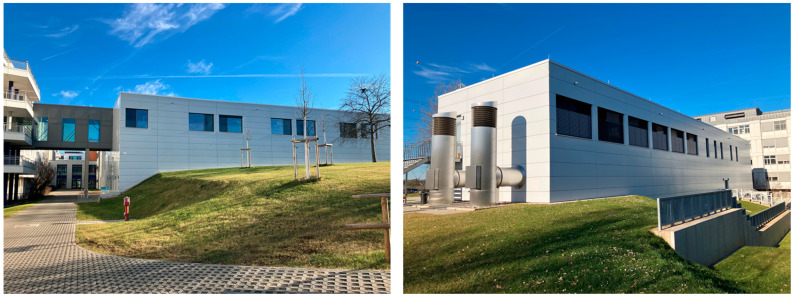
The intensive care unit building: view from the northeast (**left**) and view from the northwest (**right**). The gray bridge (**left**) forms the transition to the existing building.

The intensive care unit examined in this study is a free-standing building that was added to an existing hospital structure (Figure 1). The building consists of two floors and houses only the conservative intensive care unit. The ground floor houses the technical equipment as well as the staff changing rooms and showers. The first floor is where patient care takes place. The unit has a total of ten patient beds: 4 rooms with two beds and two isolation areas with airlocks and single beds. The unit mainly cares for patients from the field of internal medicine with a focus on cardiology. It is connected to the main building via a connecting corridor on the first floor and is separated by a restricted-access door. The building was constructed in accordance with current hygiene standards, meaning that there are no drinking water or wastewater installations in patient rooms or areas of direct medical patient care. For this reason, the station has three dirty utility rooms and one cleaning utility room where the water-carrying installations are located.

**Figure 2:**
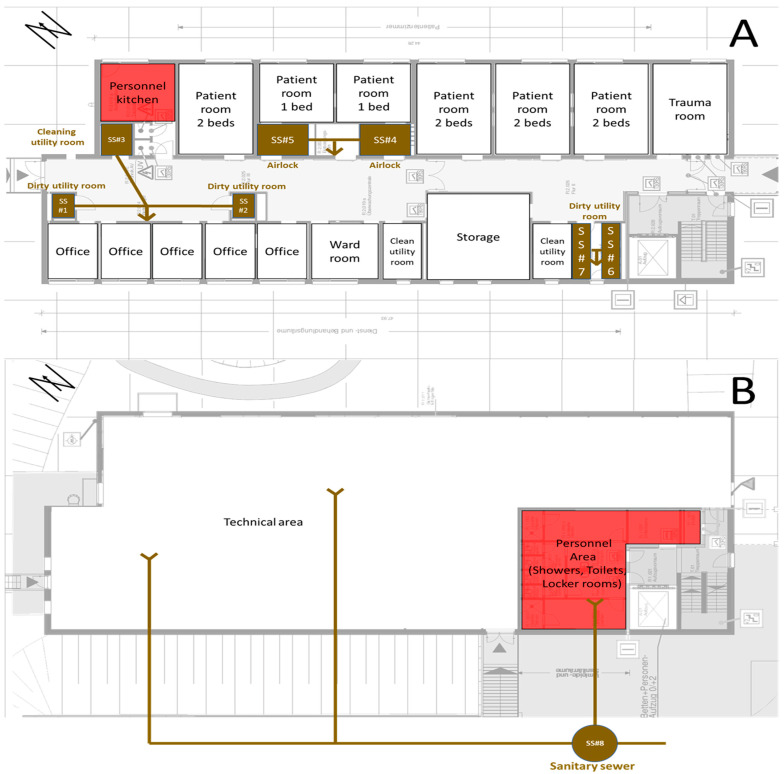
A schematic representation of the two floors of the intensive care unit. The upper floor (**A**) comprises all patient care structures. The lower floor (**B**) comprises the staff area. The sewage pipe network is shown in brown lines and arrows (“↓” meaning “downwards”, “Y” meaning “from above”) as a simplified version of the ecomaps methodology described by Kamm et al. [25]. SS: sampling site (#1 to #8). Red: personnel areas.

As a detached building, it has its own sewage system, where all effluent from the ward accumulates separately with no cross-contamination from other clinical departments before entering the hospital’s main sewage system. Figure 2 shows the water-carrying installations inside the building and the sanitary sewer outside the building from which the samples were taken.

Construction of the building’s water-carrying system was finished in April 2023. However, due to delays in the construction phase relating to radiation protection and missing IT infrastructure, further retrofitting was necessary. Therefore, medical patient operations could not begin until a year later, in April 2024. In accordance with the laws and guidelines for hygiene in drinking water installations [23,24], the building’s water-carrying system was flushed every 72 h to ensure a continuous exchange of water. This measure should prevent microbial growth and contamination through stagnation.

These special circumstances enabled controlled, pre-operational study conditions. The observation period is therefore divided into a pre-operational period and the first 12 months of patient treatment. This approach enabled direct comparison of the changes in the microbial community before and after patient-related exposure.

### 2.2. Sampling

A total of 31 samplings were performed between April 2023 and April 2025. In the twelve-month pre-operation period, nine samplings were performed. After commissioning in April 2024, 22 samplings were performed in the first 12 months of patient treatment. Samplings were taken twice weekly in the first month of operation, weekly in the second month of operation, and monthly from the third to the twelfth month of operation. Each sampling consists of seven siphon samples from SS#1 to SS#7 and a wastewater sample from SS#8.

Siphon samples were collected using sterile swabs with gel-based transport medium from SS#1 to SS#7. The wastewater samples from SS#8 were collected as scoop samples using a sampling cup attached to two ropes, which was lowered in the wastewater shaft. Because the wastewater pipes do not have a continuous flow, taps inside the building corresponding to SS#1–SS#7 were activated shortly before each sampling to ensure a continuous wastewater flow. The wastewater did not contain feces or urine, as patient excreta were disposed of separately via bedpan systems and not via the investigated sinks. At the time of sampling, no disposal of feces or urine via bedpan systems occurred to avoid fecal contamination of the sample and to ensure that the samples reflect the sink-associated wastewater and the pipe system itself. The collected wastewater was transferred to a disinfected wide-necked screw-cap bottle for transport. After each sampling event, the cup was disinfected with alcohol-based wipes.

All samples were stored at room temperature for a short period (maximum of 2 h) before being further analyzed in the laboratory.

### 2.3. Laboratory Analysis

In the laboratory, the samples were analyzed for bacteria and fungi (Enterococci, Staphylococci, *Candida*, *Acinetobacter*, *Pseudomonas*, Enterobacteriaceae, *Stenotrophomonas*, others) using a culture-based method validated for aquatic environmental samples such as wastewater [6].

#### 2.3.1. Bacterial Isolation and Cultivation

For the wastewater scoop samples, ten milliliters of each native sample were vacuum filtered onto cellulose nitrate membrane (mixed cellulose ester, diameter 50 mm, pore size 0.45 µm). Additionally, serial dilutions (1:1 to 1:10,000 in 0.9% NaCl) were prepared for the quantitative analysis. One milliliter of each dilution was plated and distributed with a Drigalski spatula on selective agar plates (CHROMAgar ESBL, CHROMAgar VRE, CHROMAgar MRSA and CHROMAgar Candida, Mast Diagnostica GmbH, Reinfeld, Germany). The filters were placed directly onto the plates. Siphon sample swabs were applied onto the plates using a three-loop smear technique.

#### 2.3.2. Subculturing and Purification of Isolates

All agar plates were incubated at 42 °C for 48 h to inhibit the growth of background flora. Growth was monitored after 24 and 48 h. All morphologically distinct looking grown colonies were counted on each plate at each dilution level to record the quantitative growth in colony-forming units. After 48 h, all morphologically distinct colonies were subcultured onto Columbia blood agar plates to obtain pure isolates for further identification and incubated at 35 ± 1 °C for 48 h [6].

#### 2.3.3. Bacterial Identification and Antimicrobial Susceptibility Testing

All of the pure subcultured isolates were subsequently identified using MALDI-TOF (Matrix-Assisted Laser Desorption/Ionization–Time-of-Flight Mass Spectrometry, Bruker Daltonics, Bremen, Germany).

Only isolates belonging to the group of nosocomial bacterial pathogens (e.g., *Enterococcus faecium*, *Enterococcus faecalis*, *Staphylococcus aureus*, *Klebsiella pneumoniae*, *Pseudomonas aeruginosa*, Enterobacteriaceae, *Candidozyma auris)* underwent antimicrobial susceptibility testing using the VITEK 2 system (bioMérieux, automated system for antibiotic susceptibility testing (AST) and identification (ID) of microorganisms) to observe the emerging of resistance for qualitative analysis. The following VITEK test cards were used: AST-N429 for *P. aeruginosa*, AST-N428 for Enterobacteriaceae, and *K. pneumoniae*, AST-N455 for Enterococci. Any ambiguous results (ATU—rea of technical uncertainty) were verified manually using ETEST gradient stripes (bioMérieux) on Müller–Hinton blood agar using a McFarland standard of 0.5 [26].

Isolates suspected to be multidrug-resistant bacteria with resistance against more than two of four main antibiotic classes of the WHO AWaRe essential “watch” list (carbapenems, third-generation cephalosporins, broad-spectrum penicillin, fluoroquinolones) underwent confirmatory manual disk diffusion testing following the EUCAST guidelines to verify the VITEK results [14,27]. In the event of a discrepancy with the MIC value determined by VITEK, the results of the manual disk diffusion test were used.

### 2.4. Statistical Analysis

The statistical analyses were performed with Stata 19 BE (Stata Corp LLC, College Station, TX, USA).

#### 2.4.1. Quantitative Analysis

The bacterial species found were categorized into eight groups for the quantitative evaluation based on a modified variant of the acronym ESKAPE [28]. The pathogens were rearranged and the acronym expanded by two groups, namely “Stenotrophomonas” and “Other” (Table 1). These groups were defined a priori. The bacteria found were then assigned to the groups based on MALDI-TOF identifications as shown in the table. No bacteria or fungi were found for ESCAPE-SO 2, 3, or 4, i.e., no *Staphylococcus aureus*, *Candidozyma auris*, or *Acinetobacter baumannii.* Taxa such as *Enterobacter* spp. were not included because they were not detected in any sample.

For each ESCAPE-SO, the median bacterial count in colony-forming units per 1 milliliter (CFU/mL) and the interquartile range were calculated by month and SS. In months with more than one sampling event, CFU values were aggregated by calculating the monthly median for quantitative analysis.

For this purpose, the mean bacterial count per species was first calculated across all evaluable dilution levels and these were totaled for each ESCAPE-SO.

A Shapiro–Wilk test was performed and found to be significant (*p* = 0.000); therefore, the statistical analysis relied on non-parametric tests. The median CFU/mL and the interquartile range of the summarized ESCAPE-SO were reported and then correlated with the start of patient treatment (Wilcoxon rank sum test, alpha = 0.05).

In addition, we verified whether the environmental factors in the samples before and during patient treatment differed significantly in order to estimate their influence on the ESCAPE-SO. For this purpose, the temperature (in °C) was measured at each sampling time point using an analog thermometer and tested with a Wilcoxon rank sum test. In months with more than one sampling event, temperature values were aggregated by calculating the monthly median temperature. The meteorological conditions were monitored qualitatively using an analog rain gauge both on the day of sampling and in the 48 h prior to sampling. Based on the criteria of the German Weather Service, a rainy day is defined as a calendar day on which a measurable amount of precipitation of at least 0.1 mm is recorded, regardless of the duration or intensity of precipitation [29]. If at least one of the days examined showed this amount of precipitation, a humid condition was recorded for the corresponding sample; arid conditions were defined as the absence of a rainy day and tested (in % of humid conditions) with a Fisher’s exact test with an alpha of 0.05 each. In months with multiple samples, a month was classified as humid if humid conditions were observed for more than 50% of the samples collected that month.

#### 2.4.2. Qualitative Analysis

Only *P. aeruginosa* was considered for the qualitative analysis. Essential “watch” antibiotics on the WHO AWaRe list [13] were selected to track the development of resistance. These are as follows:

- Carbapenems (Imipenem [IPM] and Meropenem [MPM]);
- Third-generation cephalosporins (Ceftazidime [CAZ]);
- Broad-spectrum penicillin (Piperacillin with Tazobactam [PTZ]);
- Fluoroquinolones (Ciprofloxacin [CIP]).

The other essential “watch” antibiotics were dismissed because they are not effective against *P. aeruginosa* (Azithromycin, Cefuroxime, Clarithromycin, and Vancomycin), and/or are not analyzed by the VITEK system by default (Azithromycin, Cefixime, Cefotaxime, Ceftriaxone, Clarithromycin). For the selected antibiotics, the MICs were categorized according to EUCAST as follows: “susceptible (S)”, green color; “susceptible, increased exposure (I)”, orange color; and “resistant (R)”, red color [27,30,31]. If several isolates with identical phenotypes were found in one sampling, only one isolate was reported in each case. In order to correlate the development of resistance over time with possible influencing factors, the initial detections for “I” and “R” were shown for each antibiotic group.

The hospital under investigation participates in the mandatory external quality assurance system for antibiotic consumption. This is the “Antibiotika Verbrauch Surveillance” (AVS) system of the German national public health institute, the Robert Koch Institute (RKI). The RKI-AVS uses the WHO’s ATC-DDD method [32]. The various antibiotics are classified according to their anatomical, therapeutic, and chemical properties (ATC) by a seven-digit code, e.g., J01DH02 for Meropenem. The total amount of each antibiotic used (in grams) is converted into defined daily doses (DDDs) using the substance-specific DDD values provided by the WHO Collaborating Centre for Drug Statistics Monitoring, which are updated annually [33]. The WHO defines a DDD as the “assumed average maintenance dose per day for a drug used for its main indication in adults” [32]. In the RKI-AVS, the DDDs are normalized to 100 patient days and reported as DDD rates per ward and month, allowing for standardized comparisons of antibiotic consumption across wards and over time. For the RKI-AVS, only distribution data for antibiotics were recorded, which means that the data used to calculate the DDD per antibiotic were based on the goods delivered weekly by the hospital pharmacy to the wards, without taking into account storage or actual consumption. Leistner et al. [34] have examined and discussed the disadvantages of this distribution recording system in detail and, in their work, calculated correction factors for, among other wards, the intensive care unit examined in this study. These factors are 0.75 for IPM and MPM, 1.68 for CAZ, 0.99 for PTZ, and 0.61 for CIP.

**Figure 3:**
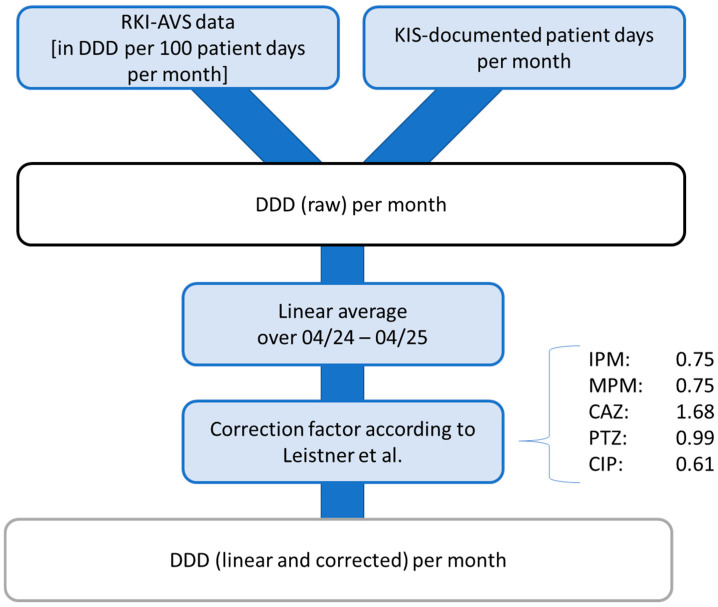
Flowchart for calculating “DDD (raw)” and “DDD (linear and corrected)”. RKI-AVS: Antibiotika Verbrauch Surveillance” of the German national public health institute, the Robert Koch Institute. DDD: defined daily dose. IPM: Imipenem. MPM: Meropenem. CAZ: Ceftazidime. PTZ: Piperacillin with Tazobactam. CIP: Ciprofloxacin. Correction factors according to Leistner et al. [34].

To counteract this known weakness in the evaluation of this study, the consumption rates reported by the RKI-AVS were therefore calculated retrospectively using the KIS-documented patient days for each month. The patient days per month were exported from the hospital information system (Nexus/KIS, Nexus AG, Villingen-Schwenningen, Germany). The linear average DDD per month over the period from April 2024 to April 2025 was then calculated for each antibiotic using this monthly “DDD (raw)”. These mean values were corrected using the correction factors described above. The values obtained in this way were accumulated for the graphical representation over the months under observation and referred to as “DDD (linear and corrected)” (Figure 3).

“DDD (raw)” and “DDD (linear and corrected)” were corrected by +1 for graphical representation. This was necessary because the range of relevant values ranged from 0 to over 500 DDDs, and a linear representation did not provide the necessary level of detail for smaller values.

## 3. Results

### 3.1. Quantitative Results of SS#8

**Figure 4:**
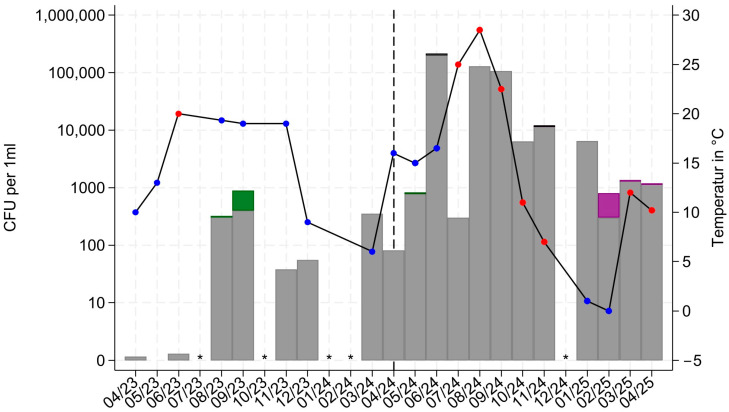
Quantitative results at SS#8 (Sanitary sewer) over the period from April 2023 to March 2025. Logarithmic Y-Scale for CFU/mL; +1 CFU/mL was added to all values. Gray bars: “Pseudomonas” (ESCAPE-SO 5). Purple bars: “Enterobacteriaceae” (ESCAPE-SO 6). Black bars: “Enterococci” (ESCAPE-SO 1). Green bars: “Others” (ESCAPE-SO 8). There was no detection of ESCAPE-SO 2, 3, 4, and 7. Black curve: temperature in °C. Blue dots: humid weather. Red dots: arid weather. Black dashed line: start of patient treatment. CFU: colony-forming units. Black asterisks: no samples taken.

The median count was 53 [0–349] CFU/mL before patient treatment and significantly higher at 8423 [3054–79,490] CFU/mL during patient treatment (*p* = 0.0224). The median temperature fell from 19 [9–19.3] °C before patient treatment to 13 [8.6–19.5] °C during patient treatment without statistical significance (*p* = 0.8657). The proportion of humid weather fell from 87.5% to 45.5% without statistical significance (*p* = 0.1470). The change in the investigated parameters over time is shown in Figure 4.

### 3.2. Qualitative Results of SS#8

The quantitative distribution of the bacterial species detected throughout the study period is shown in Table 2.

Among all identified taxa, *P. aeruginosa* was the dominant species detected in the wastewater-carrying system before and during patient treatment. For this reason, the qualitative analysis was restricted to *P. aeruginosa* as the most abundant species and a suitable indicator organism to qualitatively assess the emergence of antimicrobial resistance.

The phenotypic resistance of wildtype *P. aeruginosa* at SS#8 was stable from April 2023 to April 2024. In May and June 2024, the first two months of patient treatment, various phenotypes were observed (May: 7; June: 4), which were already associated with resistance to IPM, CAZ, and PTZ. Table 3 presents a selected subset of *P. aeruginosa* isolates. For each sampling event, the isolate showing the highest level of antimicrobial resistance was included. If multiple *P. aeruginosa* isolates with different resistance profiles were obtained from the same sample, the isolate showing either the greatest resistance or a newly observed resistance phenotype was selected.

**Figure 5:**
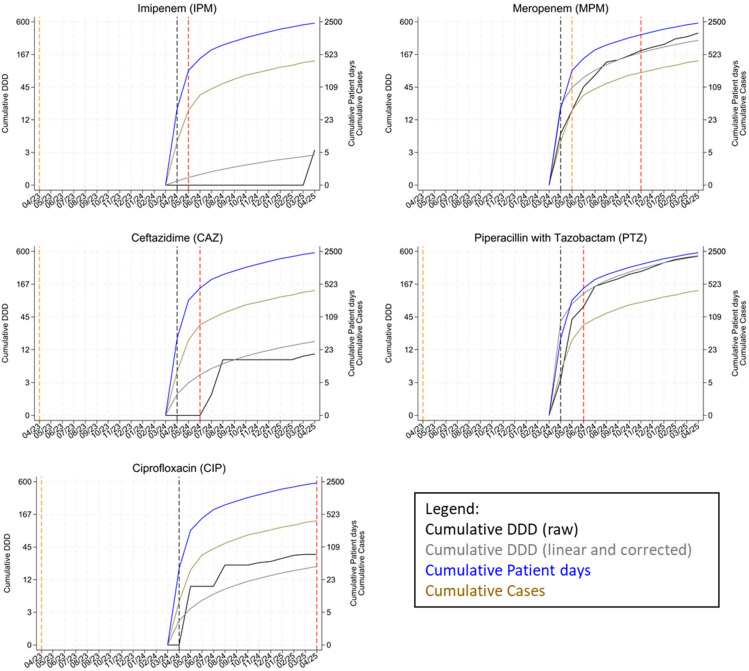
Qualitative results at SS#8 (Sanitary sewer) over the period from April 2023 to March 2025. Both *y*-axes are on a logarithmic scale; all values have therefore been adjusted upwards by 1 in the graphical presentation. Orange dashed lines: first detection of “susceptible, increased exposure”. Red dashed lines: first detection of “resistant”. Black dashed line: start of patient treatment. Black line: DDD (raw), plotted on the left *y*-axis. Gray line: DDD (linear and corrected), plotted on the left *y*-axis. Blue line: cumulative patient days, plotted on the right *y*-axis. Brown line: cumulative cases, plotted on the right *y*-axis. IPM: Imipenem. MPM: Meropenem. CAZ: Ceftazidime. PTZ: Piperacillin with Tazobactam. CIP: Ciprofloxacin. DDD: defined daily dose.

Only SS#8 (sanitary sewer) was included in further analyses (Figure 5). From April 2024 to April 2025, a total of 3.0 DDDs of IPM, 385.7 DDDs of MPM, 10.0 DDDs of CAZ, 501.2 DDDs of PTZ, and 33.7 DDDs of CIP were consumed, while 385 cases and 2353 patient days were generated.

The first detection of *P. aeruginosa* with IPM resistance in May 2024 correlated with 0.0 DDDs (raw) and 0.3 DDDs (linear and corrected) of consumed IPM, 36 cases, and 241 patient days. In the same month, “susceptible, increased exposure” was detected for the first time for MPM correlating with 17.6 DDDs (raw) and 44.5 DDDs (linear and correct) of consumed MPM.

The first detection of *P. aeruginosa* with CAZ resistance in June 2024 correlated with 0.0 DDDs (raw) and 3.9 DDDs (linear and corrected) of CAZ, 75 cases, and 433 patient days. In the same month, *P. aeruginosa* with PTZ resistance was detected for the first time and correlated with 68.1 DDDs (raw) and 114.5 DDDs (linear and corrected) of consumed PTZ.

The first detection of *P. aeruginosa* with MPM resistance in November 2024 correlated with 193.0 DDDs (raw) and 178.0 DDDs (linear and corrected) of consumed MPM, 219 cases, and 1352 patient days.

The first detection of *P. aeruginosa* with CIP resistance in April 2025 correlated with 33.7 DDDs (raw) and 20.6 DDDs (linear and corrected) of consumed CIP, 385 cases, and 2353 patient days.

### 3.3. Qualitative Results at SS#1 to SS#7

**Figure 6:**
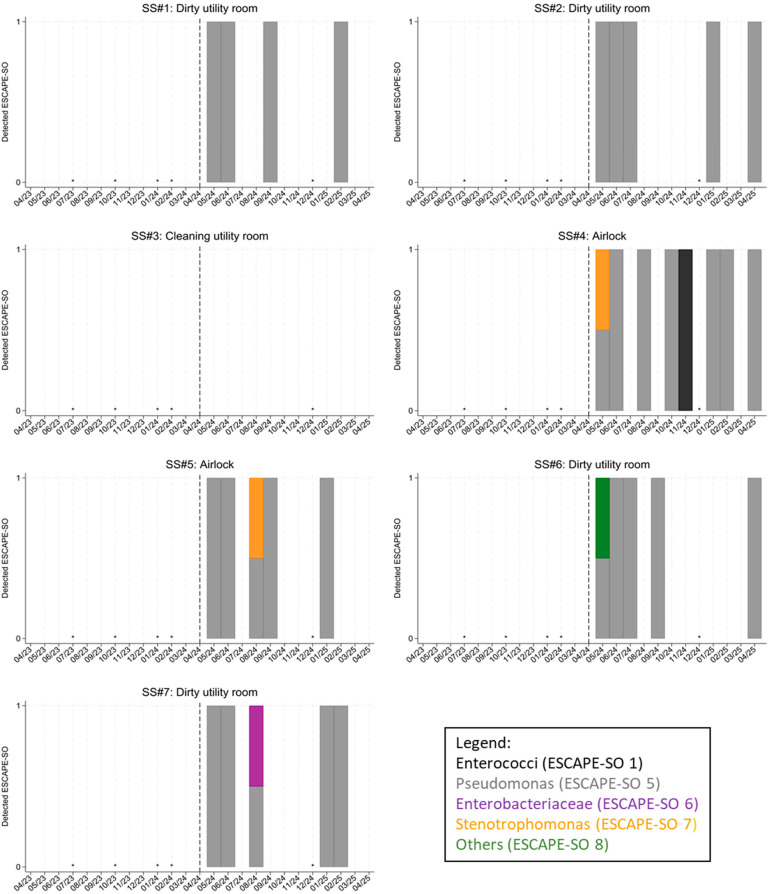
Qualitative results at SS#1 to #7 over the period from April 2023 to March 2025. Black bars: “Enterococci” (ESCAPE-SO 1). Gray bars”Pseudomonas” (ESCAPE-SO 5). Purple bars: “Enterobacteriaceae” (ESCAPE-SO 6). Orange bars: “Stenotrophomonas” (ESCAPE-SO 7). Green bars: “Others” (ESCAPE-SO 8). There was no detection of ESCAPE-SO 1, 2, 3, 4, and 7. Black dashed line: start of patient treatment. Black asterisks: no samples taken.

Before the start of patient treatment, no bacteria or fungi were detected at any of the sampling sites SS#1 to #7. After the start of patient treatment, bacteria and/or fungi were detected at several sampling sites (Figure 6). At SS#1, “Pseudomonas” (ESCAPE-SO 5) was detected. At SS#2, “Pseudomonas” (ESCAPE-SO 5) was detected. No bacterial or fungal growth was detected at SS#3. At SS#4, “Pseudomonas” (ESCAPE-SO 5) was detected. Furthermore, “Enterococci” (ESCAPE-SO 1) and “Stenotrophomonas” (ESCAPE-SO 6) were detected. At SS#5, “Pseudomonas” (ESCAPE-SO 5) was detected, as well as “Stenotrophomonas” (ESCAPE-SO 6). At SS#6, “Pseudomonas” (ESCAPE-SO 5) was detected, as well as “Others” (ESCAPE-SO 8). At SS#7, “Pseudomonas” (ESCAPE-SO 5) was detected, as well as “Enterobacteriaceae” (ESCAPE-SO 6).

Table 4 shows the phenotypes for the other SSs. Before patient treatment, no resistant *P. aeruginosa* was detected. In May and June 2024, resistant strains were detected at all SSs. Further resistance was discovered at SS#2, #4, and #7 over time.

## 4. Discussion

This study aimed to characterize the quantitative and qualitative changes in the microbial community of a newly built internal medicine intensive care unit (ICU) wing following the start of patient treatment.

### 4.1. Evaluation of Generated Data

#### 4.1.1. Quantitative Data

The quantitative analysis showed an increase in the bacterial and fungal species examined in the sanitary sewer (SS#8) after the start of patient treatment (median quantity 53 [0–349] CFU/mL before patient treatment and 8423 [3054–79,490] CFU/mL during patient treatment). This increase was not related to humidity (*p* = 0.1470), nor to temperature (*p* = 0.8657). The change can therefore be explained by the start of patient treatment (*p* = 0.0224). SS#1 to #7 (siphons) showed no bacterial or fungal growth in the pre-operation period but demonstrated a quantitative increase after the start of patient treatment (except SS#3). As these sampling points were located inside the building, weather and temperature factors were not taken into account for further analyses.

#### 4.1.2. Qualitative Data

Qualitative analysis of the sanitary sewer (SS#8) showed wildtype *P. aeruginosa* in all samples during the pre-operational period. This wildtype *P. aeruginosa* showed a phenotypically stable resistance pattern (no acquired resistance, consistently susceptible to increased exposure to Piperacillin, Piperacillin Tazobactam, Ceftazidime, Cefepime, Imipenem, and Ciprofloxacin, and susceptible to Meropenem). During the twelve months of patient treatment, a rapid increase in the frequency of resistant isolates was observed. Nine months after the start of patient operation, *P. aeruginosa* isolates from the sanitary sewer exhibited resistance to three of four main antibiotic classes of the WHO AWaRe essential “watch” list (carbapenems, third-generation cephalosporins, broad-spectrum penicillin, fluoroquinolones). After twelve months, resistance to fluoroquinolones was also detected for the first time.

The first signs of resistance appeared within the first month after the start of patient treatment (IPM), the second month (PTZ and CAZ), the seventh month (MEM), and the twelfth month (CIP). Patient days vary greatly between the antibiotic groups. The amount of antibiotics consumed therefore appears to play a greater role than the number of patient days. This is plausible, as not every patient receives antibiotics and antibiotic-free patient days should therefore have no effect on resistance.

In addition, there were very different DDD thresholds at the points in time when the first instances of resistance were detected. Resistance to IPM occurred within the first month after the start of patient treatment, although the ward’s data do not reflect high consumption. One possible explanation for this phenomenon is the underlying resistance mechanism in *P. aeruginosa*. Carbapenem exposure, including MPM, is known to select for mutations and regulatory changes affecting the OprD porin, AmpC, and RND efflux pumps, which frequently manifest first as an IPM-resistant phenotype. Resistance to MPM is harder to achieve compared to IPM, because OprD is the main uptake channel for IPM and only a minor route for MPM. Loss or downregulation of OprD produces high-level resistance to IPM with comparatively modest increases in MPM MICs. High MPM consumption, such as on the ward, can therefore lead to early detectable resistance to IPM, before resistance to MPM is detectable [35,36]. Early IPM resistance without a clinical consumption correlate could therefore be interpreted as a sensitive indicator of early adaptions of the microbiome to high MPM consumption. The same phenomenon can be observed with CAZ: here, too, resistance develops at an early stage, even though consumption data indicate that CAZ was hardly used at all. The underlying mechanisms therefore appear to play a key role in the development of resistance, depending on the fitness costs for *P. aeruginosa*. Resistance (such as IPM and CAZ) can develop quickly, while other mechanisms generate high fitness costs and require higher selection pressure before they develop (such as MPM) [37]. Consequently, a low DDD value does not necessarily lead to low selection pressure. High selection pressure can also arise from low amounts of antibiotics or other substances if the threshold for the development of a resistance mechanism is low for the microorganism. The required selection pressure therefore depends on the mutation architecture of the target organism. Thus, it can be concluded that a high DDD does not necessarily lead to immediate resistance if the resistance mechanism is complex. A simple linear DDD–resistance correlation does not appear to exist, but even moderate antibiotic consumption can generate measurable resistance in the wastewater microbiome within a few weeks, as this study indicates.

The qualitative analysis of SS#1 to #7 showed mixed results. While SS#2, #4, and #7 showed development of resistance over time, this was not the case for SS #1, #5, and #6; SS#3 did not show growth of any bacteria at all. The design and length of the walkways seem to play a major role in explaining these findings (Figure 2): SS#1 is the most distant dirty utility room and is therefore probably less used than the others—maybe even not used at all. SS#3 is a clean utility room and not expected to be used for patient fluid disposal. SS#6 is the clean side of a dirty utility room and also not expected to be used for patient fluid disposal. Only the missing resistance development at SS#5 cannot be explained without digging deeper into the clinical data. It could be that fewer patients were on antibiotic therapy in this isolation room than in the neighboring room that corresponds to SS#4. However, as this information is not relevant to the aim of this study, no further analysis was carried out here and the findings were valued as random.

### 4.2. Strengths Compared to Similar Studies

Compared with previous studies on hospital wastewater, this is the first study to include a defined pre-operational monitoring period of hospital wastewater.

Nolasco-Rojas et al. (2025) investigated the release of antibiotic-resistant bacteria and β-lactam antibiotics through hospital wastewater [20]. Their work highlights the importance of proper management of hospital wastewater, as they identified a wide variety of resistant pathogens in hospital wastewater. However, the most notable difference is that their study did not include a pre-operational observation phase. In contrast, Nolasco-Rojas et al. initiated sampling only after the facility was in full operation. Our study’s approach allowed for the establishment of a true microbial baseline prior to patient admission.

Stoesser et al. (2024) conducted a longitudinal genomic study of hospital wastewater across six wards over a 12-month period [38]. Their study focused on the dissemination of blaKPC-producing Enterobacterales. In contrast, this study focused on quantitative growth after the beginning of patient treatment and on *P. aeruginosa* as a key environmental opportunist. Our study did not include parallel patient screening. Both studies point out the role of hospital wastewater as a reservoir for antibiotic-resistant bacteria. Stoesser et al. demonstrated the genetic diversification of established resistance within active hospital wastewater systems, whereas this study provides complementary insight into how resistant bacteria emerge during the earliest stages of system use.

### 4.3. Limitations

There are three relevant limitations of this work. The first and major limitation of this study is that a genomic similarity analysis of *P. aeruginosa* isolates from both wastewater and siphons was not performed. Due to the lack of external funding, molecular typing approaches such as whole-genome sequencing or PCR-based fingerprinting were not feasible within the scope of this study. Consequently, it cannot be conclusively determined whether the observed increase in resistance resulted from in situ evolution of wildtype *P. aeruginosa* under resistance pressure by antibiotic residues or from survival of “fitter” resistant strains from patients that re-colonized the system. The first explanation is supported by the correlation between antibiotic consumption and the development of resistance, as shown in Figure 5, as well as the missing data of any patients who have been treated for a resistant *P. aeruginosa*-associated infection. In addition, an evaluation of routine hygiene swab data as part of routine microbiological screening (at admission and once a week during stay for each patient) did not indicate the coincidental introduction of a highly resistant *P. aeruginosa* strain associated with a specific patient. These observations support the interpretation that the increasing occurrence of resistance traits is unlikely to be explained by repeated introduction of distinct resistant clones. However, the detection of different phenotypes of resistant isolates per sampling event indicates that both explanations might be right and no definitive conclusions regarding clonal persistence can be drawn. Therefore, further research, especially future studies incorporating molecular typing, is needed here.

The second limitation of this study is the irregular and decreasing sampling frequency over time. A total of 31 samples were collected between April 2023 and April 2025, including 9 pre-operation and 22 post-commissioning samples. After commissioning, samples were collected twice weekly during the first month, weekly during the second month, and monthly thereafter. While this design allowed for detailed monitoring of early microbial shifts, the reduced frequency later in the study may have limited the detection of short-term variations or transient resistance events. However, this irregularity is unlikely to have significantly affected the overall findings.

The third limitation of this study is the lack of follow-up samples after the 12-month observation period of patient treatment. It therefore remains unclear whether the observed changes in the wastewater microbiome are also long-term trends. Future studies should include longer-term monitoring to better understand the persistence and progression of resistance in hospital wastewater systems.

### 4.4. The Impact on the Environment

The findings of this study also have broader implications. Hospital wastewater serves as an interface between clinical antimicrobial use, environmental dissemination, and public health risks. This study’s findings demonstrate that hospital activity can rapidly promote the development of antimicrobial resistance within wastewater microbial communities. Such resistance spreads from the hospital sewage system to the environment.

The HyReKA Consortium (2022) showed that wastewaters exposed to hospital effluents show a considerably higher contamination by Gram-negative bacteria with antibiotic resistance [39]. Antibiotic-resistant bacteria from hospital wastewater in Germany were even detected in surface waters, bathing waters, drinking water reservoir systems, and mussels [40,41,42]. Hospitals are therefore major emitters of antibiotic-resistant bacteria into the environment. To avoid the spread of antibiotic residues and antibiotic-resistant bacteria into these water bodies, retention and targeted treatment of hospital effluents are needed to limit the environmental dissemination of antimicrobial resistance.

Retention strategies such as antibiotic stewardship programs can reduce consumption [18], but even moderate consumption of antibiotics is reflected in the wastewater microbiome through the rapid development of resistance, as this study shows. Therefore, further strategies are needed to prevent AMR from reaching the environment.

Municipal wastewater treatment plants are unable to achieve 100 percent purification of wastewater. The effluent from sewage treatment plants therefore continues to contain ARB and resistance genes, which are then discharged into surface waters [43]. In order to treat all remaining substances using state-of-the-art technology, sewage treatment plants require what is known as a “fourth treatment stage”. Not every method of the fourth treatment stage is suitable for effectively reducing ARB. Activated carbon filtration, which is often used, is not sufficiently effective for reducing the microbiological load in wastewater [44]. Ultrafiltration, ozonation, and a combination of ozonation and UV irradiation show the most significant reduction or elimination performance, with ultrafiltration achieving the most effective reduction [44,45].

One way to prevent the spread of ARB into the environment at an early stage could be the decentralized treatment of hospital wastewater at the point of origin. Hospitals represent defined, clearly identifiable point sources; targeted treatment prior to discharge into municipal wastewater treatment plants would not only relieve the burden on wastewater treatment plants but also prevent horizontal exchange of resistance genes within them [46]. This approach would also be consistent with the polluter paying for environmental protection measures, but it would be necessary to discuss who would ultimately bear the costs in the case of hospital wastewater.

## 5. Conclusions

This study demonstrates that the start of patient treatment in a newly opened intensive care wing led to a rapid and significant increase in both microbial load and the emergence of antimicrobial resistance in hospital wastewater, confirming that hospital activity drives early and measurable changes in the wastewater microbiome. The occurrence of resistant *P. aeruginosa* increased within months after the start of clinical operations; however, there were not clear signs of its development despite intangible descriptive connections to the consumed DDDs of antibiotics and the generated cases or patient days. Based on the overall results, stricter antibiotic stewardship, targeted interventions at the point of wastewater discharge, and consideration of advanced wastewater treatment technologies should be implemented to limit the spread of resistant pathogens into the environment.

## Figures and Tables

**Table 1:** Aggregation of bacterial species into eight groups for quantitative analysis: ESCAPE-SO.

Group	ESCAPE-SO	Plate	Bacterial Species
1	Enterococci	VRE	*Enterococcus casseliflavus* *Enterococcus faecalis* *Enterococcus faecium*
2	Staphylococci	MRSA	None
3	Candida	Candida	None
4	Acinetobacter	ESBL	None
5	Pseudomonas	ESBL	*Pseudomonas aeruginosa* *Pseudomonas nitroreducens* *Pseudomonas oleovorans* Other *Pseudomonas* spp.
6	Enterobacteriaceae	ESBL	*Citrobacter freundii* *Escherichia coli* *Klebsiella pneumoniae* Other *Citrobacter* spp.
7	Stenotrophomonas	ESBL	*Stenotrophomonas maltophilia*
8	Other	ESBL	All other spp.

**Table 2:** Quantitative distribution of the bacterial (and fungal) species in SS#8 (sanitary sewer) throughout the study period. *N*: total number of observed months. *n*: months with detection of specific ESCAPE-SO group. IQR: interquartile range. *: statistically significant Wilcoxon rank sum test.

ESCAPE-SO	Before Patient Treatment (*N* = 8)		During Patient Treatment (*N* = 12)		*p*
	*n*/*N* (%)	Median [IQR]	*n*/*N* (%)	Median [IQR]	
Enterococci	0 (0.0%)	0 [0–0]	3 (25.0%)	0 [0–0.5]	0.3860
Staphylococci	0 (0.0%)	0 [0–0]	0 (0.0%)	0 [0–0]	
Candida	0 (0.0%)	0 [0–0]	0 (0.0%)	0 [0–0]	
Acinetobacter	0 (0.0%)	0 [0–0]	0 (0.0%)	0 [0–0]	
Pseudomonas	7 (88.5%)	45.5 [0.2–330.0]	12 (100.0%)	3860 [541.9–59,350.0]	0.0014 *
Enterobacteriaceae	0 (0.0%)	0 [0–0]	5 (52.7%)	0 [0–9.5]	0.1022
Stenotrophomonas	0 (0.0%)	0 [0–0]	0 (0.0%)	0 [0–0]	
Others	2 (25%)	0 [0–0.1]	1 (8.3%)	0 [0–0]	0.2947

**Table 3:** Minimal inhibitory concentrations (MICs) for *P. aeruginosa* at SS#8 (Sanitary sewer). IPM: Imipenem. MPM: Meropenem. CAZ: Ceftazidime. PTZ: Piperacillin with Tazobactam. CIP: Ciprofloxacin. Green field: susceptible. Yellow field: susceptible, increased exposure. Red field: resistant. Black dashed line: start of patient treatment.

Isolate	Date	IPM	MPM	CAZ	PTZ	CIP
2	04/23	2	0.25	2	8	0.12
F9-1	04/23	2	0.25	2	8	0.12
7	06/23	2	0.25	2	8	0.12
17	08/23	2	0.25	2	8	0.12
26	09/23	2	0.25	2	8	0.12
32	11/23	2	0.25	2	8	0.12
45	03/24	2	0.25	2	4	0.25
61	04/24	2	0.25	2	8	0.12
72	05/24	32	4	2	8	0.12
81	05/24	2	0.25	2	8	0.12
93	05/24	1	0.25	8	16	0.12
108	05/24	2	0.25	8	16	0.12
143	05/24	4	0.25	2	4	0.12
164	05/24	2	1	8	16	0.5
183	05/24	2	0.25	2	16	0.12
204	06/24	2	0.25	2	8	0.12
213	06/24	2	0.5	16	128	0.12
229	06/24	2	0.25	2	8	0.12
252	06/24	16	4	2	8	0.12
270	07/24	0.5	0.25	2	4	0.06
288	08/24	2	0.25	2	8	0.12
313	09/24	2	0.25	2	4	0.12
332	09/24	2	4	2	128	0.12
350	10/24	2	1	2	8	0.25
366	11/24	16	16	2	32	0.25
400	01/25	8	16	64	64	0.12
425	02/25	8	16	64	128	0.12
452	03/25	8	4	32	32	0.12
475	04/25	16	16	16	16	0.12
465	04/25	16	16	16	16	4

**Table 4:** Minimal inhibitory concentrations (MICs) for *P. aeruginosa* at SS#1 to #7, excluding #3 (cleaning utility room, no detection of any bacteria). IPM: Imipenem. MPM: Meropenem. CAZ: Ceftazidime. PTZ: Piperacillin with Tazobactam. CIP: Ciprofloxacin. Green field/S: susceptible. Yellow field/I: susceptible, increased exposure. Red field/R: resistant.

Sampling Site (SS)	Phenotype	IPM	MPM	CAZ	PTZ	CIP
SS#1 Dirty utility room	Wildtype (05/24)	I	S	I	I	I
	First I	-	-	-	-	-
	First R	-	-	-	-	-
SS#2 Dirty utility room	Wildtype (05/24)	I	S	I	I	I
	First I	-	01/25	-	-	-
	First R	-	-	08/24	08/24	-
SS#4 Airlock	Wildtype (06/24)	I	S	I	I	I
	First I		11/24	-	-	-
	First R	11/24	-	-	07/24	-
SS#5 Airlock	Wildtype (05/24)	I	S	I	I	I
	First I	-	-	-	-	-
	First R	-	-	-	-	-
SS#6 Dirty utility room	Wildtype (04/25)	I	S	I	I	I
	First I	-	-	-	-	-
	First R	-	-	-	-	-
SS#7 Dirty utility room	Wildtype (05/24)	I	S	I	I	I
	First I	-	-	-	-	-
	First R	08/24	04/25	01/25	08/24	-

## Data Availability

The dataset is available on request from the authors.

## Abbreviations

The following abbreviations are used in this manuscript:

AMR	Antimicrobial resistance
ARB	Antimicrobial-resistant bacteria
ARG	Antimicrobial resistance gene
ATC	Anatomic–therapeutic–chemical properties
AVS	Antibiotika Verbrauch Surveillance (Antibiotic Consumption Surveillance System)
CAZ	Ceftazidime
CFU	Colony-forming units
CIP	Ciprofloxacin
DDD	Defined Daily Dose
I	Susceptible, increased exposure
IQR	Interquartile range
IPM	Imipenem
MIC	Minimal inhibitory concentration
ml	Milliliter
MPM	Meropenem
PTZ	Piperacillin Tazobactam
R	Resistant
RKI	Robert Koch Institute
spp.	Species
S	Susceptible
SS	Sampling site
WHO	World Health Organization
